# An Appreciation of "Ierne," by a Nurse with Service and Decorations

**Published:** 1918-10-26

**Authors:** 


*To the Editor of* THE HOSPITAL.

### Tradition or Progress?

### An Appreciation of "Ierne," by A Nurse with Service and Decorations.

*To the Editor of* THE HOSPITAL.

SIR,—Tradition shrouds our minds even in these enlightened days. It obscures, smothers, stifles, and deters many efforts at reform. Tradition has formed a very thick encrustation over the Nursing Profession, difficult to pierce through even by the most ambitious partisans of progress. It is called by many of its adherents "noble ideals," and apt to be misconstrued as the true fabric composing the tenets of the great profession.

To any effort at progress and reform we hear from all sides cries and exhortations to remember ideals. The College of Nursing at its inception and inauguration received much of this chorus, as it was supposed that the College would sweep away all the ideals and the traditions ever created by nurses and nursing. But it is hard to find the true ideals and live up to them apart from the obscurity of tradition with which they are enveloped, for it is traditional to dare to raise a voice in protest, traditional to remain inarticulate, inert, apparently indifferent, to carry on uncomplainingly, and endure steadfastly because of those traditions, so-called ideals, which are brought forward and used as arguments against the fostering of this new feeling, of a desire for equity and re-establishment, which of late has been agitating our ranks.

There has arisen a writer having the courage of conviction in bringing to the light of day and to the sense of those closely concerned the many points open to doubtful question, for long existent in the nursing world, and has stirred up a storm of comment, because many of the old traditions are severely criticised. It cannot be adequately recorded, the admiration and appreciation due to this bold and impartial writer "Ierne," whose articles are followed with zest. It would be good if such writings appeared in the public Press, so that the man and woman in the street should read and know some of the conditions that women work under in this the noblest, but badly organised profession. Hitherto each institution has been a law unto itself, having diverse and manifold laws, according to its own lights. Every nursing home, nursing association has individual authority beginning and ending within it. No legislation or central authority control these latter for the protection of the nurse from exploitation.

From the salary offered in so many hospitals for skilled and trained work, equal to that of a medium grade of domestic service, also with precisely the same subsistence conditions, though with less liberty and freedom, we have the nurse working in "private," under the co-operative system, which, in some instances, extracts the high premium of twenty per cent. on all earnings, but giving and affording no protection or guarantee in the case of bad debts, so frequently occurring in the floating population of popular resorts. Thus this class of worker often suffers severe losses.

The nursing home, privately owned. Its wealthy patients paying excessive fees. Its nurses, with their long hours of heavy stair work, receiving £30 a year. Yet tradition and ideals keep these workers struggling on, voiceless and inarticulate.

The whole fabric of the nursing system is changing. It has almost ceased to be a vocation, and is becoming a profession. As a profession it demands the rights and recognition of a profession, establishment upon a sound basis of construction, controlled by one deciding governing authority. The status elevated by drawing a good class of member, and clear decided rules laid down for adequate remuneration after training; cramping institutional environment removed and intellectual development encouraged.

Nurses need an intrepid champion, someone to watch the trend of events for them. They need someone to chip away the incubus of tradition that obscures the commonsense out of otherwise sensible people. The medical profession will not support them, for they fear trespass upon their own prerogatives. It needs the impartial looker-on, with no axe to grind, to take up their cause—one who has lived and worked amongst them and has now stepped aside.

Nurses will never support themselves, they are already too overburdened with their mission and their work. Striving to live up to ideal, battling for ever against the narrowed vision, narrowed perceptions of the traditions of the past, outworn under the disguising cloak of charity, that charity that is ever a bad paymaster.

Times are changing. The whole system and fabric is changing. Gone are the women who took nursing as a vocation or a religion. All honour to them.

A new generation has sprung up, with demands proportionate to the times. Into it has come the fresh, energetic, emancipated, practical inquiring worker, cognisant of her worth and value; determined that it shall not be squandered uselessly, but giving of her best where it meets with appreciation and acknowledgment; seeking justice and equity and proper recognition for a science applied to a most necessitous cause, and adequate remuneration for skilled services rendered thereto.

That the old system of charity unfairly distributed and extorting the best years of life service in a blind execution of duty, because it is a traditional ideal to do so, without just compensation, be assigned to a receding past, together with the " halo argument." To strive and hope for better and freer conditions, fairer repayment, for the uplifting of the grand profession to higher levels, when it will merit the acknowledgment of its aims, not only of the needy and indigent, but of the intellectual, the thinkers, the pioneers of true reform. A. H. I.

